# Antimicrobial Copper Cold Spray Coatings and SARS-CoV-2 Surface Inactivation

**DOI:** 10.1557/adv.2020.366

**Published:** 2020-12-01

**Authors:** Bryer C. Sousa, Danielle L. Cote

**Affiliations:** Materials Science and Engineering, Worcester Polytechnic Institute, Worcester, MA USA

**Keywords:** Antiviral Materials, Contact Killing/Inactivating Surfaces, Antimicrobial Coatings, Copper, Cold Spray, COVID-19, SARS-CoV-2, Antipathogenic Coatings

## Abstract

This article contextualizes how the antimicrobial properties and antipathogenic contact killing/inactivating performance of copper cold spray surfaces and coatings and can be extended to the COVID-19 pandemic as a preventative measure. Specifically, literature is reviewed in terms of how copper cold spray coatings can be applied to high-touch surfaces in biomedical as well as healthcare settings to prevent fomite transmission of SARS-CoV-2 through rapidly inactivating SARS-CoV-2 virions after contaminating a surface. The relevant literature on copper-based antipathogenic coatings and surfaces are then detailed. Particular attention is then given to the unique microstructurally-mediated pathway of copper ion diffusion associated with copper cold spray coatings that enable fomite inactivation.

## INTRODUCTION

The objective of this review article is to situate the way in which the antipathogenic properties and antimicrobial contact killing/inactivating performance of copper cold spray coatings and surfaces can be extended to the COVID-19 pandemic as a preventative measure against transmission. Specifically, literature is reviewed from a materials performance and mechanistic perspective on how copper cold spray coatings are successfully able to be utilized as high-touch surfaces in biomedical as well as healthcare settings as an antimicrobial and viricidal material solution for enhanced prevention of fomite transmission of SARS-CoV-2 through rapidly inactivating SARS-CoV-2 virions after infecting a surface. For example, nursing homes, medical facilities, public transportation, and schools have become high traffic focal points for the spread and transmission of the SARS-CoV-2 virus during the current pandemic. Such settings house a significant volume of high touch surfaces on which the SARS-CoV-2 virus has been shown to be able to remain active and transmissible. By way of refitting the high touch surfaces of the most vulnerable locations and organizations with such antiviral copper cold spray coatings, our functionalized coatings would be able to contribute to the mitigation and prevention of SARS-CoV-2 infection.

## FOMITE TRANSMISSION OF VIRAL PATHOGENS AND SARS-COV-2

In 2017, Xiao et al. made sure to remind the scientific community that "the distinct potential for a resurgence of SARS," or a previously unseen SARS-like coronavirus, outbreak remained even though new outbreaks of the first SARS virus have yet to resurface after 2004 [1]. Xiao et al. went on to analytically investigate how fomite transmission of SARS infections were found to play a *non-negligible* role in pathogen transmission throughout a simulated hospital setting. The study by Xiao et al. is only one of a number of similar works attesting to the fact that preventative measures need to be taken to minimize the risk of infection associated with touching an infected surface when SARS-based coronavirus outbreaks occur [2–5]. Otter et al. found that SARS-CoV, MERS-CoV, and the influenza virus was shed from an infected community member within a hospital-like setting at concentrations that are much greater than the dose required for infection [6]. Otter et al. went on to note that typical hospital surfaces were reportedly found to be infected and discussed the potential of fomite transmission and subsequent surface-to-hand and hand-to-mucous membrane inoculation of said viral pathogens in [6]. With particular attention being given to SARS-CoV-2 and similar viruses, Castaño et al. focused on disinfection approaches to inhibit surface-contact mediated fomite transmission in [7]. Additional works of scholarship worth consideration include [4,8–14].

## COPPER AS AN OLIGODYNAMIC ANTIMICROBIAL MATERIAL

Numerable means of antimicrobial copper-containing materials and coatings production have been reported upon to date. Given the resurgence in reported research concerned with this topic, only a handful of current research articles are situated, as well as discussed below, within the array of antipathogenic copper surfaces, which have been reported upon to date. Accordingly, a hybrid poly-(lactide-co-glycolide), copper-oxide nanoparticle containing composite, and nanofiber-based scaffolding, was developed by Haider et al. through the use of electrospinning in [15]. Antibacterial testing demonstrated inhibited bacterial growth of Gram-positive and Gram-negative strains. Another composite based material, comprised of a copper-zirconium-aluminium metallic glass, was developed by Villapun et al. [16,17]. Villapun et al. found that the crystallinity of the metallic glass composite increased proportionally with copper content. Villapun et al.'s antibacterial testing analysis identified the $Cu_{56}Zr_{38.7}Al_{5.3}$ metallic glass composite composition as the antipathogenic material stoichiometry that achieved the maximal antibacterial performance after one hour of exposure to Gram-negative *E. coli* and Gram-positive *B. subtilis*. Each of the copper-based metallic glass composites were also found to completely eliminate the bacteria tested through a time-kill approach after no more than 250 minutes of direct contact and surface exposure.

Ciacotich et al. published an analysis of the antibacterial efficacy of an alloyed copper coating with silver as the alloying element in [18]. To perform a proper investigation of the antipathogenic performance, the copper-silver coating was subjected to testing conditions, which were defined by an EPA protocol of relevance, wherein a bacterial biofilm was imposed upon the surface of the alloyed copper-silver coating. Ciacotich et al. found that a Gram-positive bacterial biofilm was completely killed within 5 minutes of exposure time. Continued consideration of Ciacotich et al.'s hypothesis shows that the microorganism contact killing activity and behaviour associated with copper-silver coatings is a multifactorial/multivariate and complex process, which depends upon copper ion diffusion, bacterial cell oxidation, local variations in pH, and more. Turning towards commercially pure copper as the antimicrobial material of particular focus, Kocaman et al. produced biocidal wire arc sprayed copper coatings using a twin wire arc spray gun and a stainless-steel substrate surface [19]. Moreover, Kocaman et al. characterized the antibacterial efficacy of copper coatings using a wire arc spray deposition process after exposure to various bacterial pathogens. The pathogens explored consisted of Methicillin-resistant *S. aureus* (MRSA), *P. aeruginosa*, Vancomycin-resistant *Enterococcus*, *E. coli*, and *S. aureus*.

Kocaman et al. found that *E. coli*, *S. aureus*, and *P. aeruginosa* "vanished from the surface" after 15 minutes of exposure to the wire arc sprayed copper coating. Intriguingly, the Vancomycin-resistant *Enterococcus* and MRSA superbugs required more time for complete contact killing and inactivation to occur. At 15 minutes, 100% of the MRSA was killed (initially) whereas only 96.11% of the Vancomycin-resistant *Enterococcus* was passivated. At 1 hour, 98% of the Vancomycin-resistant *Enterococcus* was killed while 98.3% of the MRSA was killed. Finally, at 2 hours, 99.7% of the Vancomycin-resistant *Enterococcus* was killed while 100% of the MRSA was killed. Kocaman et al. concluded by way of reiterating the fact that "no difference was observed in biocidal performance" between Gram-positive and Gram-negative bacteria. Kocaman et al. also provided a reasonable hypothesis as to why the superbugs required greater exposure times for complete contact killing based upon the fact that superbugs have been found to have "thicker cell membranes, possibly causing a decrease in the rate of ion diffusion through the cell wall." It is the opinion of the authors of the present review article that the hypothesis provided by Kocaman et al. deserves continued investigation in future work. While discussion could continue, the final copper-containing approach, which will be noted herein before transitioning to SARS-CoV-2 and viral contact inactivation, concerns the work of Muralidharan et al. Specifically, a sulphonated poly-(ether-ether-ketone)-copper film for antimicrobial applications was detailed by Muralidharan et al. [20].

Another recent work of scholarship documented the use of Luminore CopperTouch™ coatings to inactivate SARS-CoV-2 on coated surfaces [21]. Unfortunately, the research by Mantlo et al. does not appear to delve too deeply into the realm of mechanisms associated with copper-mediated contact inactivation of SARS-CoV-2. Regardless, the Luminore CopperTouch™ surface was found to inactivate 99% of the SARS-CoV-2 titters within 2 hours while also inactivating 99.9% of the *Ebola* as well as *Marburg* viruses in that period of time as well. Consistent with our own claim that cuprous oxide ($Cu_2O$) is likely to be just as effective as pure copper in diffusing the copper ions needed for viral contact inactivation[22], recent work undertaken at Virginia Polytechnic Institute and State University has identified another copper-based coating that can also rapidly inactivate SARS-CoV-2 too[23]. Still, one of the most promising aspects of copper cold spray antipathogenic coatings relative to the coatings presented by Behzadinasab et al. and

Mantlo et al. is the likelihood of even greater inactivation rates below 1-hour of exposure time given the dynamically recrystallized and severely plasticly deformed microstructure, which greatly enhances ion diffusivity.

## ANTIMICROBIAL COPPER COLD SPRAY COATINGS

While antimicrobial copper coatings have been presented to the scientific community with greater regularity since the 20$^{th}$ century, one of the first focused publications was published by Champagne and Helfritch [24]. Champagne and Helfritch's research illustrated how copper cold spray surfaces achieved greater antibacterial performance as a contact killing surface in comparison with two alternative copper surfaces, which were generated using plasma spray and wire arc spray. The demonstration by Champagne and Helfritch was achieved by way of inoculating MRSA on the three thermally sprayed surfaces for 2 hours, based on an EPA protocol of relevance, and the MRSA cells that survived were assayed. In comparison with the percentage of MRSA that survived 2 hours of direct contact and exposure to the plasma sprayed copper surface, which was greater than 10% when normalized against a stainless-steel control surface, the copper cold spray surface killed more than 99.999% of the inoculated antibiotic resistant MRSA.

Soon thereafter, Champagne and Helfritch reiterated their original findings [24] in subsequent papers explicitly focused upon highlighting the unique applications well-suited for cold spray materials consolidation. That being said, Champagne and Helfritch did not stop exploring the antimicrobial efficacy associated with copper cold spray surfaces after publishing their original "proof of concept" study using MRSA as the test case. Rather, they subsequently partnered with academic researchers (Sundberg et al.) to demonstrate the way in which more rapid contact killing/inactivation rates can be achieved by utilizing a novel nanostructured spray-dried pure copper feedstock powder instead of the conventionally gas-atomized powder typically used in copper cold spray deposition [25]. At the same time, Sundberg et al. extended the realm of microbes from one bacterial species (MRSA) to that of viral pathogens via *Influenza A* as the test case as well. During Sundberg et al.'s initial venture into the realm of explicitly antiviral copper cold spray coatings, a 99.3% reduction of active Influenza A virions was achieved in accordance with the aforementioned EPA-inspired 2 hour exposure time of the virus to the nanostructured copper cold spray coatings. With respect to the conventional copper cold sprayed coating, a 97.7% reduction of active *Influenza A* virions was also observed.

Thereafter, publications focusing on various material aspects such as surface roughness, surface species and surface chemistry, corrosion, and microstructure, were pursued by Sundberg et al. [26–30] Around the same time, Champagne, Sundberg, and Helfritch, co-authored a more focused document that reformulated their respective mechanistic framework for interpreting the postulated reasons as to why antimicrobial copper cold spray coatings antipathogenic efficacies outperform many alternative surface engineering solutions [31]. Not long after, a holistic framework for understanding the antipathogenic performance unique to copper cold spray coatings was presented by Sousa et al. [22]

Beyond the work of Champagne et al., Sundberg et al., and Sousa et al., additional researchers have also started to explore and consider antimicrobial copper-based cold spray coatings. In so far as the literature is concerned, Vilardell et al.'s review article considered most of the antimicrobial cold spray applications reported prior to 2015. Since that time, Rutkowska-Gorczyca investigated the microstructure of an antibacterial copper and titanium dioxide composite coatings produced using low pressure cold spray in [32]. Sanpo et al. analyzed a copper and zinc oxide composite cold sprayed coating for the purpose of contact killing and prohibiting *C. marina* bacterial attachment to maritime vessel surfaces [33]. Vucko et al. demonstrated antifouling capacities of high-density polyethylene "[metalized] with copper powder using cold spray" in [34,35]. El-Eskandrany et al. explored the use of a copper-based alloyed metallic glass powder feedstock for antibacterial cold spray application [36]. Additional work by da Silva et al. [37] resulted in self-sanitizing copper cold spray coatings that "completely inhibited after 10 [minutes] of direct contact between the bacteria and the coating surface."

## MECHANISM OF ENHANCED ANTIVIRAL PERFORMANCE

Having established the non-negligible transmission pathway associated with SARS-CoV-2 and other viral pathogens via fomites wherein surface-to-hand and hand-to-mucous membrane inoculation occurs; copper as the most oligodynamic, antimicrobial, and naturally occurring elemental metal; and the application of copper cold spray materials consolidations and surfaces as an antipathogenic biomaterial, another critical question remains to adequately appreciate antimicrobial copper cold spray. Explicitly, one may wonder the following: "Why is antimicrobial copper cold spray able to achieve enhanced antiviral performance?" Irrespective of ribonucleic acid and/or nucleoprotein damage, membrane and/or membrane protein damage, or reactive oxygen species (ROS) formation and pathogen interaction is/are the primary mechanism underpinning copper's capacity to inactivate or kill pathogens in contact with a surface, the ability of a copper surface to readily diffuse copper ions to the microbe in contact with the surface dictates if any of the currently proposed damage pathways can be actualized.

That is to say, in most of the pathogen-copper killing/inactivation interactions reported upon to date, such as genomic damage, membrane disruption and damage, ROS, or atomic copper ion speciation [38], microstructural and physical pathways for copper ion diffusion to the infectious agent from the copper material must be achieved. As briefly discussed earlier, Champagne et al. attributed the unique antipathogenic performance of copper cold spray coatings to the "extreme work hardening and correspondingly high dislocation density within the deposit… and ionic diffusion occurs principally through these dislocations…" associated with the supersonic particle consolidation process in [24]. Succeeding articles were published by Champagne et al., among others who collaborated with Champagne, in an effort to support and substantiate Champagne et al.'s dislocation-driven hypothesis. This was pursued by way of utilizing a relation between hardness and dislocation density, such that higher hardness's were entertained as an indicator of increased antipathogenic performance for cold sprayed copper surfaces [25]. However, in 2019 and 2020, Sousa et al. began to further analyse the microstructures of antipathogenic copper cold sprayed materials to investigate the suitability of Champagne et al.'s dislocation driven copper ion diffusion framework for assessing the increased contact killing/inactivation rates for both viral and microbial pathogens relative to non-cold spray materials/solutions. As a result, the most current assessment and research provides readers with a detailed look into the role such material defects, i.e., dislocations, maintain relative to the role of grain-boundary mediated copper ion diffusion [22].

## CONCLUDING REMARKS AND FUTURE OUTLOOK

Considering the successful inactivation of Influenza A virions after exposure to copper cold spray coatings, in accordance with EPA evaluation protocols, and research demonstrating inactivation of SARS-CoV-2 after exposure to a conventional and less viricidal copper-based material (relative to copper cold spray surfaces), copper cold spray materials consolidations could quickly be optimized as a focused mitigation strategy for the COVID-19 pandemic. Deploying antipathogenic copper cold spray surfaces in the fight against SARS-CoV-2 and COVID-19 will simultaneously provide continued public health security by way of mitigating the magnitude of future pandemics through the prevention of fomite transmission from these coatings and microstructurally unique copper material.

Regardless of the fact that fomite transmission “isn’t thought to be the main way the virus [SARS-CoV-2] spreads,” the CDC recognized “it may be possible that a person can get COVID-19 by touching a surface or object that has the virus [SARS-CoV-2] on it and then touching their own mouth, nose, or possibly their eyes.” Research published by Han et al. also attested to the need for antiviral materials in high-touch contact containing environments in the fight against SARS-CoV-2 [39], wherein Han et al. stated that “SARS-CoV-2 can be transmitted by droplets and contact. A study in South Korea showed that many environmental surfaces of patients with MERS were contaminated by MERS-CoV, and virus RNA was detected from environmental surfaces within 5 days after the last positive PCR of patients’ respiratory samples.” The World Health Organization (WHO) also spoke to the legitimacy associated with fomite transmission of the novel SARS-CoV-2 coronavirus as well. Copper cold spray is a material advancement as an antimicrobial and preventative measure consistent with the statements from the CDC and WHO as well as the research performed by Han et al. and many others.
